# A Novel Approach for Predicting Disease-lncRNA Associations Based on the Distance Correlation Set and Information of the miRNAs

**DOI:** 10.1155/2018/6747453

**Published:** 2018-06-26

**Authors:** Haochen Zhao, Linai Kuang, Lei Wang, Zhanwei Xuan

**Affiliations:** ^1^College of Information Engineering, Xiangtan University, Xiangtan 411105, China; ^2^Key Laboratory of Intelligent Computing & Information Processing, Xiangtan University, Xiangtan 411105, China

## Abstract

Recently, accumulating laboratorial studies have indicated that plenty of long noncoding RNAs (lncRNAs) play important roles in various biological processes and are associated with many complex human diseases. Therefore, developing powerful computational models to predict correlation between lncRNAs and diseases based on heterogeneous biological datasets will be important. However, there are few approaches to calculating and analyzing lncRNA-disease associations on the basis of information about miRNAs. In this article, a new computational method based on distance correlation set is developed to predict lncRNA-disease associations (DCSLDA). Comparing with existing state-of-the-art methods, we found that the major novelty of DCSLDA lies in the introduction of lncRNA-miRNA-disease network and distance correlation set; thus DCSLDA can be applied to predict potential lncRNA-disease associations without requiring any known disease-lncRNA associations. Simulation results show that DCSLDA can significantly improve previous existing models with reliable AUC of 0.8517 in the leave-one-out cross-validation. Furthermore, while implementing DCSLDA to prioritize candidate lncRNAs for three important cancers, in the first 0.5% of forecast results, 17 predicted associations are verified by other independent studies and biological experimental studies. Hence, it is anticipated that DCSLDA could be a great addition to the biomedical research field.

## 1. Introduction

For long time, RNA was just considered to be transcriptional noise and intermediary between a DNA sequence and its encoded protein [1, 2]. However, sequence analyses point out that more than 98% of the human genome does not encode protein sequences [3]. Furthermore, increasing studies based on biological experiments have indicated that ncRNAs play important roles in numerous critical biological processes such as chromosome dosage compensation, epigenetic regulation, and cell growth [4]. In particular, the lncRNAs, as a class of important ncRNAs with a length more than 200 nucleotides [5], have been found to be associated with a wide range of human diseases, such as breast cancer [6], colorectal cancer [7], lung cancer [8], and cardiovascular diseases [9]. Hence, the study of finding novel disease-lncRNA associations has captured the attention of a lot of researchers and has been considered as one of the hottest topics in the research fields of diseases and lncRNAs. The identification of disease-lncRNA association can not only accelerate the understanding of human complex disease mechanism at the lncRNA level, but also serve as a biomarker identification for human disease diagnosis, treatment, and prevention [10]. So far, a lot of studies have generated a large amount of lncRNAs related biological data about sequence, expression, function, and so on [11–13]. However, compared with the rapidly increasing number of newly discovered lncRNAs, only few known lncRNA-disease associations have been reported. Hence, it is challenging and urgently needed to develop efficient and successful computational approaches to predict potential lncRNA-disease associations. In recent years, some computational methods have been proposed to predict novel lncRNA-disease associations, which can significantly decrease the time and cost of biological experiments by calculating the association probability of lncRNA-disease pairs. For example, Chen G et al. presented the first prediction method (genomic locus based) and constructed a lncRNA-disease association database as well [14]. Liang et al. proposed a genetic mediator and key regulator model to unveil the subtle relationships between lncRNAs and lung cancer. Liu et al. developed a computational framework to accomplish this by combining human lncRNA expression profiles, gene expression profiles, and human disease-associated gene data. Applying this framework to available human long intergenic noncoding RNAs (lincRNAs) expression data, Chen et al. developed a semi-supervised learning method based on framework of Laplacian Regularized Least Squares, LRLSLDA, to infer potential lncRNA-disease associations which did not need negative samples and could obtain a reliable AUC of 0.7760 in the leave-one-out cross-validations [15]. In 2014, Sun et al. constructed a lncRNA functional similarity network and applied random walk with restart (RWR) to infer potential lncRNA-disease associations [16]. In the same year, Li et al. presented a bioinformatics method based on genomic location to predict the lncRNAs associated with vascular disease [17]. Then, Zhao et al. developed a computational method based on the naïve Bayesian classifier to identify cancer-related lncRNAs by integrating genome, regulome, and transcriptome data [18]. In 2015 Zhou et al. proposed a novel rank-based method named RWRHLDA to prioritize candidate lncRNA-disease associations by integrating miRNA-associated lncRNA-lncRNA crosstalk network, disease-disease similarity network, and known lncRNA-disease association network into a heterogeneous network and implemented a random walk with restart on the newly generated heterogeneous network [19].

Nowadays, with advent of many biological datasets, such as LncRNADisease [14], lncRNAdb [20], and NONCODE [13], the number of lncRNA-disease associations is still very limited. In 2015, Chen developed a method, named HGLDA, based on the information of miRNA [21], which predicted lncRNA-disease associations by integrating disease-miRNA associations with lncRNA-miRNA interactions and did not rely on known lncRNA-disease associations. Different from the method of HGLDA proposed by Chen et al., in this article, on the basis of experimentally reported lncRNA-disease associations collected from the HMDD database [22] and miRNA-lncRNA associations collected from the starBase database [23], a novel model based on distance correlation set is developed to predict potential lncRNA-disease associations by integrating known lncRNA-miRNA associations and known miRNA-disease associations. Compared with HGLDA, the advantage of DCSLDA lies in the introduction of the similarity of disease pairs and lncRNA pairs and distance correlation set. In addition, to optimize the prediction performance of DCSLDA, new methods to calculate the similarity of disease-disease pairs and lncRNA-lncRNA pairs are developed simultaneously. Finally, to evaluate the prediction performance of DCSLDA, LOOCV is implemented on the basis of the known lncRNA-disease associations and known lncRNA-cancer associations separately, and simulation results demonstrate that DCSLDA is superior to the state-of-the-art methods and can achieve a reliable AUC of 0.8517 in the LOOCV when the pregiven threshold parameter *r* is set at 6. Additionally, to further evaluate the prediction performance of DCSLDA, case studies of breast cancer, colorectal cancer, and lung cancer are implemented for DCSLDA; as a result, among the first 0.5% of predictive results, 9, 6, and 2 predicted potential associations are confirmed by recent experimental reports, respectively. Hence, considering the excellent prediction performance of DCSLDA, it is obvious that DSCLDA can become a useful and efficient computational tool for biomedical researches.

## 2. Materials and Methods

### 2.1. Disease-miRNA Associations

We downloaded known disease-miRNA associations from the Human MicroRNA Disease Database (HMDD) in July 2017 (see Supplementary file 1), which included 10381 experimentally verified disease-miRNA associations (including 572 miRNAs and 383 diseases). After merging miRNAs which produce the same mature miRNA and eliminating duplicate data, we obtained* dataset1* including 5430 disease-miRNA associations (including 383 human diseases and 495 lncRNAs). Let *D* be the number of different diseases and* M1* be the number of different miRNAs collected from the* dataset1*, respectively, *S*_*D*_ = {*d*_1_, *d*_2_,…, *d*_*D*_} represent the set of these *D* different diseases, and *S*_*M*1_ = {*m*1_*D*+1_, *m*1_*D*+2_,…, *m*1_*D*+*M*1_} represent the set of these* M1* different miRNAs; then for any given *d*_*i*_ ∈ *S*_*D*_ and *m*1_*j*_ ∈ *S*_*M*1_, we can define the* Association Strong Correlation *(*ASC1*) between *d*_*i*_ and *m*1_*j*_ as follows:

(1) $$\begin{array}{c} ASC1\left(d_{i},{m1}_{j}\right)=\left\{\begin{array}{cc} 1, & \text{If }d_{i}\;\;is\;\;related\;\;to\;\;{m1}_{j}\;\;in\;\;the\;\;dataset1\\ 0, & otherwise. \end{array}\right. \end{array}$$

### 2.2. miRNA-lncRNA Associations

We downloaded known miRNA-lncRNA associations dataset from starBase v2.0 dataset in July 2017, which provided the most comprehensive experimentally confirmed lncRNA-miRNA interactions based on large scale CLIP-seq data. After data preprocessing (including elimination of duplicate values, erroneous data, disorganized data, and so on),* dataset2* (including 10195 lncRNA-miRNA associations, 275 miRNAs, and 1127 lncRNAs) was obtained from the starBase v2.0 (see Supplementary file 2). Let* M2* be the number of different miRNAs and *L* be the number of different lncRNAs collected from the* dataset2*, *S*_*M*2_ = {*m*2_1_, *m*2_2_,…, *m*2_*M*2_} represent the set of these* M2* different miRNAs, and *S*_*L*_ = {*l*_*M*2+1_, *l*_*M*2+2_,…, *l*_*M*2+*L*_} represent the set of these *L* different lncRNAs; then, for any given *m*2_*i*_ ∈ *S*_*M*2_ and *l*_*j*_ ∈ *S*_*L*_, we can define the* ASC2* between* m2*_i_ and *l*_*j*_ as follows:

(2) $$\begin{array}{c} ASC2\left({m2}_{i},l_{j}\right)=\left\{\begin{array}{cc} 1, & \text{If }{m2}_{i}\;\;is\;\;related\;\;to\;\;l_{j}\;\;in\;\;the\;\;dataset2\\ 0, & otherwise. \end{array}\right. \end{array}$$

### 2.3. lncRNA-Disease Associations

In order to evaluate the performance of DCSLDA, the newly lncRNA-disease associations were downloaded from LncRNADisease database, which integrated more than 1000 lncRNA-disease entries and 475 lncRNA interaction entries, including 321 lncRNAs and 221 diseases from ~500 publications. In this dataset, after duplicate associations and the lncRNA-disease associations involved in either diseases or lncRNAs which were not contained in the* dataset1* or* dataset2* were removed, 203 high-quality lncRNA-disease associations were obtained finally (see Supplementary file 3).

### 2.4. Disease Functional Similarity Based on miRNAs

**Figure 1:**
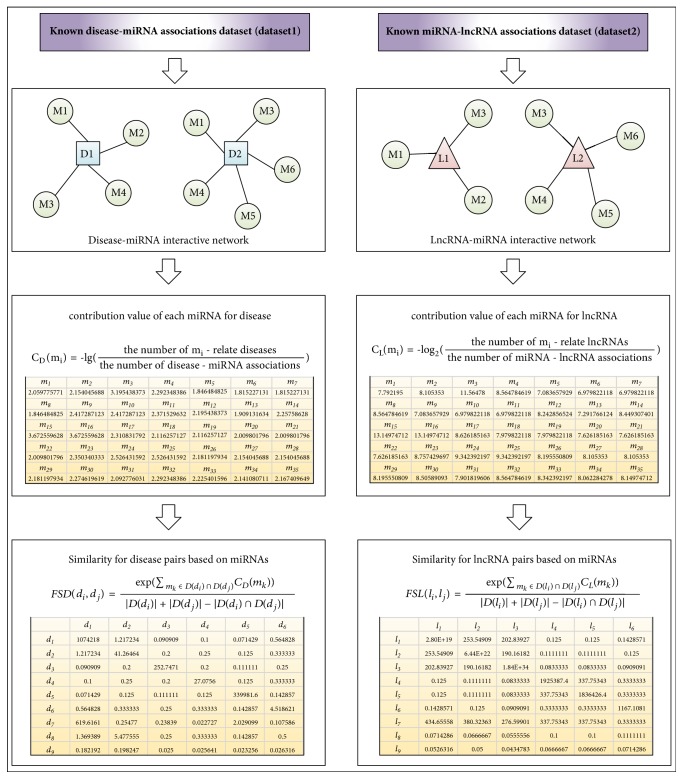
The flowchart of functional similarity calculation based on information of miRNA includes three steps: (1) constructing known disease-miRNA association and miRNA-lncRNA association network respectively; (2) obtaining contribution of each miRNA; (3) calculating functional similarity for diseases and lncRNAs, respectively.

For calculating the functional similarity between diseases, we introduced the concept of social network. In the social network, for any two nodes, we can calculate the similarities between them by comparing and integrating the similarities of nodes associated with these two nodes. In this section, based on the assumption that similar diseases tend to show a similar interaction and noninteraction pattern with the miRNAs, we calculated the disease similarity in the disease-miRNA interactive network. As illustrated in Figure 1, the calculation procedures of disease functional similarity based on miRNAs include 3 steps. First, we constructed miRNA-disease interactive network from known miRNA-disease associations (*dataset1*), whose topology can be abstracted as an undirected graph *G*_1_ = (*V*_1_, *E*_1_), where *V*_1_ = *S*_*D*_ ∪ *S*_*M*1_ = {*d*_1_, *d*_2_,…, *d*_*D*_, *m*1_*D*+1_, *m*1_*D*+2_,…, *m*1_*D*+*M*1_} is the set of vertices, *E*_1_ is the set of edges, and, for any two nodes *a*, *b* ∈ *V*_1_, there is an edge between *a* and *b* in *E*_1_, if and only if there are *a* ∈ *S*_*D*_, *b* ∈ *S*_*M*1_, and *ASC*1(*a*, *b*) = 1. However, since different miRNA terms in the* dataset1* may relate to different numbers of diseases, it is not suitable to assign the same contribution value to different miRNAs. Hence, we define the contribution value of each miRNA as follows:

(3) $$\begin{array}{c} C_{D}\left(m_{i}\right)=-\mathrm{lg}\left(\frac{the\;\;number\text{ of}\;\;m_{i}-related\;\;edges\;\;in\;\;E_{1}}{the\;\;number\text{ of}\;\;all\;\;edges\;\;in\;\;E_{1}}\right). \end{array}$$

Finally, we defined the functional similarity between diseases *di* and *dj* by integrating the miRNAs related to *di*, *dj*, or both of them as follows:

(4) $$\begin{array}{c} FSD\left(d_{i},d_{j}\right)=\frac{\exp\sum_{m_{k}\in \left(D\left(d_{i}\right)\cap D\left(d_{j}\right)\right)}C_{D}\left(m_{k}\right)}{\left|D\left(d_{i}\right)\right|+\left|D\left(d_{j}\right)\right|-\left|D\left(d_{i}\right)\cap D\left(d_{j}\right)\right|} \end{array}$$

where* FSD* is the disease functional similarity matrix calculated based on miRNA and *D*(*d*_*i*_) and *D*(*d*_*j*_) are the number of d_i_ related edges and d_j_ related edges in E_1_, respectively. As an example, in Figure 1, there is* FSD*  (*d*_1_, *d*_2_) = exp⁡(*C*_*D*_(*m*_1_) + *C*_*D*_(*m*_3_) + *C*_*D*_(*m*_4_))/(4 + 5 − 3).

### 2.5. lncRNA Functional Similarity Based on miRNAs

Based on the assumption that similar lncRNAs tend to show a similar interaction and noninteraction pattern with the miRNAs, we can calculate the lncRNA similarity in the lncRNA-miRNA interactive network. Similar to the calculation procedures of disease functional similarity, first, we constructed lncRNA-miRNA interactive network from known lncRNA-miRNA associations (*dataset2*), whose topology can be abstracted as an undirected graph *G*_2_ = (*V*_2_, *E*_2_), where *V*_2_ = *S*_*M*2_ ∪ *S*_*L*_ = {*m*2_1_, *m*2_2_,…, *l*_*M*2+1_, *l*_*M*2+2_,…, *l*_*M*2+*L*_} is the set of vertices, *E*_2_ is the set of edges, and, for any two nodes *a*, *b* ∈ *V*_2_, there is an edge between *a* and *b* in *E*_2_, if and only if there are *a* ∈ *S*_*M*2_, *b* ∈ *S*_*L*_, and *ASC*2(*a*, *b*) = 1. Then, considering the number of lncRNA-miRNA associations, we defined the contribution value of each miRNA as follows:

(5) $$\begin{array}{c} C_{L}\left(m_{i}\right)=-\log_{2}\left(\frac{the\;\;number\text{ of}\;\;m_{i}-related\;\;edges\;\;in\;\;E_{2}}{the\;\;number\text{ of}\;\;all\;\;edges\;\;in\;\;E_{2}}\right). \end{array}$$

Additionally, we defined the functional similarity between lncRNA *l*_*i*_ and *l*_*j*_ by integrating the miRNAs related to *l*_*i*_, *l*_*j*_, or both of them as follows:

(6) $$\begin{array}{c} FSL\left(l_{i},l_{j}\right)=\frac{\exp\sum_{m_{k}\in \left(D\left(l_{i}\right)\cap D\left(l_{j}\right)\right)}C_{L}\left(m_{k}\right)}{\left|D\left(l_{i}\right)\right|+\left|D\left(l_{j}\right)\right|-\left|D\left(l_{i}\right)\cap D\left(l_{j}\right)\right|} \end{array}$$

where* FSL* is the disease functional similarity matrix calculated based on miRNA and *D*(*l*_*i*_) and *D*(*l*_*j*_) are the number of *l*_*i*_ related edges and *l*_*j*_ related edges in *E*_2_, respectively.

### 2.6. Method for Predicting Potential Association between lncRNAs and Diseases

**Figure 2:**
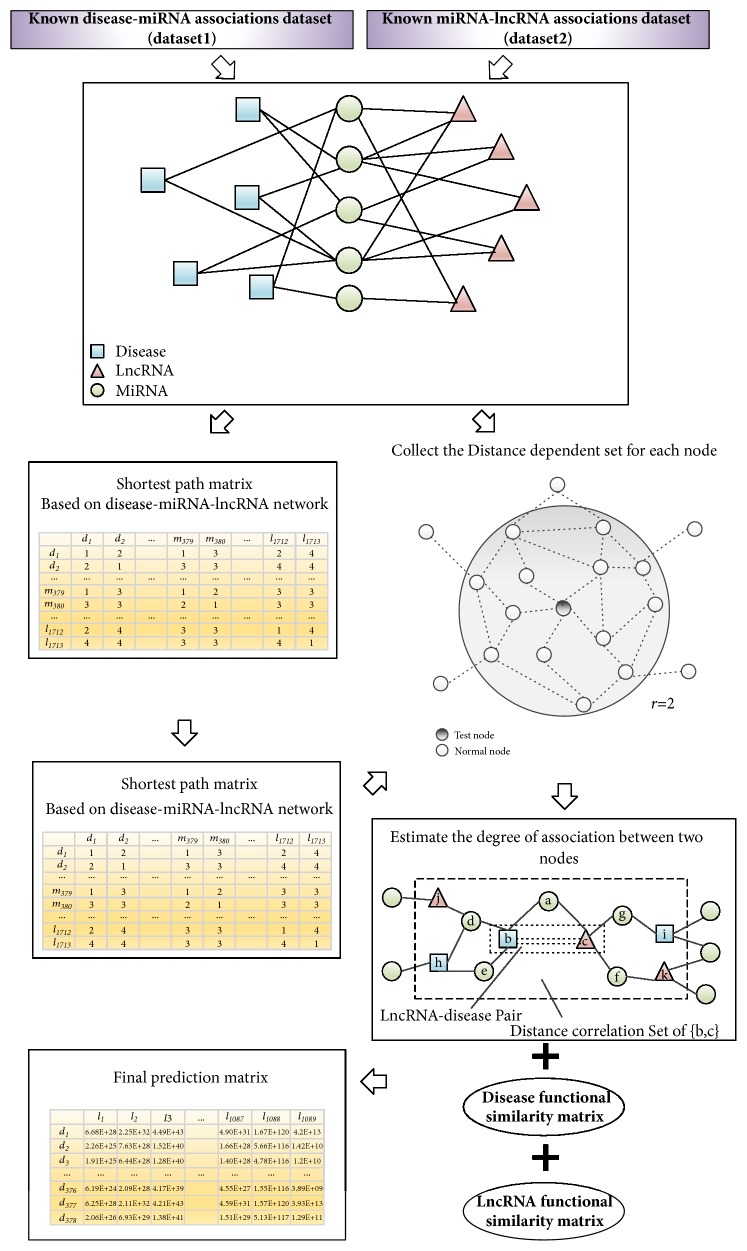
The procedures of DCSLDA.

Based on the assumptions that similar diseases tend to show a similar interaction and noninteraction pattern with the miRNAs and similar miRNAs tend to show a similar interaction and noninteraction pattern with the lncRNAs, we proposed a novel model, DCSLDA, based on miRNAs and distance correlation set to predict potential disease-lncRNA associations. As illustrated in Figure 2, the procedures of DCSLDA consist of the following 6 major steps.

**Step 1 (construction of the disease-miRNA-lncRNA interaction network).**

On the basis of the above descriptions and letting *M* = *M*1∩*M*2, we can construct a disease-miRNA-lncRNA interaction network based on* dataset1* and* dataset2*, whose topology can be abstracted to an undirected graph *G*_3_ = (*V*_3_, *E*_3_), where *V*_3_ = *S*_*D*_ ∪ *S*_*M*_ ∪ *S*_*L*_ = {*d*_1_, *d*_2_,…, *d*_*D*_, *m*_*D*+1_, *m*_*D*+2_,…, *m*_*D*+*M*_, *l*_*D*+*M*+1_, *l*_*D*+*M*+2_,… , *l*_*D*+*M*+*L*_} is the set of vertices, *E*_3_ is the edge set of *G*_3_, and ∀*l*_*i*_ ∈ *L*, *m*_*j*_ ∈ *M*, *d*_*k*_ ∈ *D*. There is an edge between *l*_*i*_ and *m*_*j*_ in *E*_3_, if and only if the lncRNA *l*_*i*_ relates to the miRNA *m*_*j*_. Moreover, there is an edge between *m*_*j*_ and *d*_*k*_ in *E*_3_, if and only if the miRNA *m*_*j*_ is related to the disease *d*_*k*_. Then, for any given *a*, *b* ∈ *V*_3_, we can define the* ASC3* between a and b as follows:

(7) $$\begin{array}{c} ASC3\left(a,b\right)=\left\{\begin{array}{cc} 1, & \text{If }there\;\;exists\;\;an\;\;edge\;\;between\;\;a\;\;and\;\;b\;\;in\;\;the\;\;E_{3}\\ 0, & otherwise. \end{array}\right. \end{array}$$

In addition, although we did not use any known disease-lncRNA associations, the diseases and lncRNAs can still be linked by integrating edges between diseases node and miRNAs node and edges between miRNAs nodes and lncRNAs nodes in the *G*_3_.

**Step 2 (construction of the* Adjacency Matrix *based on the disease-miRNA-lncRNA interactive network).**

We can construct a (*D* + *M* + *L*)×(*D* + *M* + *L*) dimensional* Adjacency Matrix *(*AM*) based on the disease-miRNA-lncRNA interactive network as follows:

(8) $$\begin{array}{c} AM\left(i,j\right)\left\{\begin{array}{c} ASC3\left(d_{i},d_{j}\right),\text{ if }i\in \left[1,D\right],\;\;j\in \left[1,D\right].\\ ASC3\left(d_{i},m_{j}\right),\text{ if }i\in \left[1,D\right],\;\;j\in \left[D,D+M\right].\\ ASC3\left(d_{i},l_{j}\right),\text{ if }i\in \left[1,D\right],\;\;j\in \left[D+M,D+M+L\right].\\ ASC3\left(m_{i},d_{j}\right),\text{ if }i\in \left[D,D+M\right],\;\;j\in \left[1,D\right].\\ ASC3\left(m_{i},m_{j}\right),\text{ if }i\in \left[D,D+M\right],\;\;j\in \left[D,D+M\right].\\ ASC3\left(m_{i},l_{j}\right),\text{ if }i\in \left[D,D+M\right],\;\;j\in \left[D+M,D+M+L\right].\\ ASC3\left(l_{i},d_{j}\right),\text{ if }i\in \left[D+M,D+M+L\right],\;\;j\in \left[1,D\right].\\ ASC3\left(l_{i},m_{j}\right),\text{ if }i\in \left[D+M,D+M+L\right],\;\;j\in \left[D,D+M\right].\\ ASC3\left(l_{i},m_{j}\right),\text{ if }i\in \left[D+M,D+M+L\right],\;\;j\in \left[D+M,D+M+L\right] \end{array}\right. \end{array}$$

where *i* ∈ [1, *D* + *M* + *L*] and *j* ∈ [1, *D* + *M* + *L*].

**Step 3 (construction of the shortest distance matrix based on the disease-miRNA-lncRNA interactive network).**

Let *r* be a pregiven positive integer; then we can obtain *r* matrixes such as *AM*^1^, *AM*^2^, …, *AM*^*r*^ based on the* Adjacency Matrix*. Then, we can construct a (*D* + *M* + *L*)×(*D* + *M* + *L*) dimensional Shortest Path Matrix (*SPM*) as follows:

(9) $$\begin{array}{c} SPM\left(i,j\right)=\left\{\begin{array}{cc} 0, & \text{if }{AM}^{r}\left(i,j\right)=0\\ 1, & \text{if }AM\left(i,j\right)=1\\ k, & otherwise \end{array}\right. \end{array}$$

where *i* ∈ [1, *D* + M + *L*], *j* ∈ [1, *D* + M + *L*], *k* ∈ [2, *r*], and *k* satisfies *AM*^*k*^(*i*, *j*) ≠ 0 while *AM*^1^(*i*, *j*) = *AM*^2^(*i*, *j*) = ⋯ = *AM*^*k*−1^(*i*, *j*) = 0.

**Step 4 (collection of the* distance correlation sets* for nodes in the interactive network).**

In *G* = (*V*, *E*), let *V* = {*d*_1_, *d*_2_,…, *d*_*D*_, *m*_*D*+1_, *m*_*D*+2_,…, *m*_*D*+*M*_, *l*_*D*+M+1_, *l*_*D*+M+2_,…, *l*_*D*+M+*L*_} = {*v*_1_, *v*_2_,…, *v*_*D*_, *v*_*D*+1_, *v*_*D*+2_,…, *v*_*D*+*M*_, *v*_*D*+M+1_ , *v*_*D*+M+2_,…, *v*_*D*+M+*L*_}; then for each node *v*_*i*_ ∈ *V*, we can obtain its distance correlation set *DCS*_*i*_ according to the shortest distance matrix as follows:

(10) $$\begin{array}{c} {DCS}_{i}=\left\{v_{j}\;|\;r\geq SPM\left(i,j\right)>0,\;\;i\neq j\right\}. \end{array}$$

**Figure 3:**
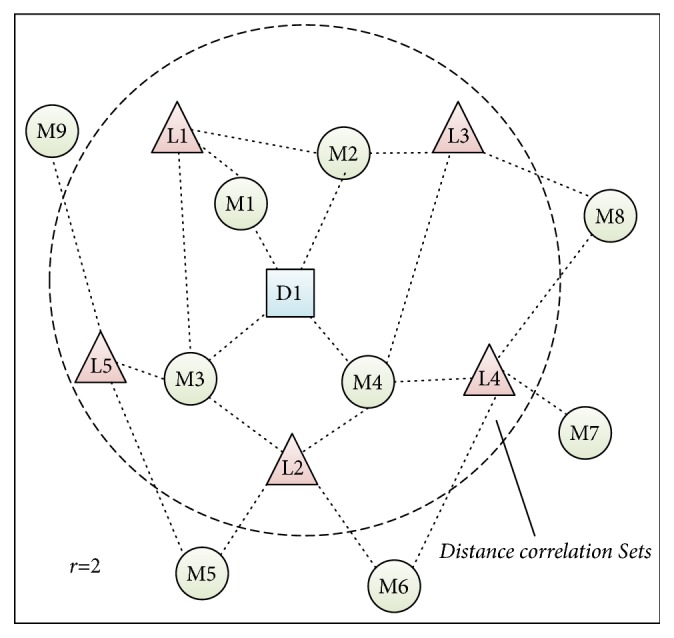
Distance correlation set of D1 with r=2.

For instance, in the disease-miRNA-lncRNA interaction network illustrated in Figure 3, supposing that we hope to collect the* DCS*_D1_, then according to the above description, we can easily know that the* distance correlation sets *of* D1* will be {M1, M2, M3, M4, L1, L2, L3, L4, L5} when *r* = 2.

And thereafter, for any given node *v*_*j*_ ∈ *DCS*_*i*_, where *j* ≠ *i*, we can compute the distance correlation coefficient *P*(*i*, *j*) between the node *v*_*i*_ and *v*_*j*_ as follows:

(11) $$\begin{array}{c} P\left(i,j\right)=P\left(v_{i},v_{j}\right)=\left\{\begin{array}{cc} 1-\frac{SPM\left(i,j\right)}{r+1}, & \text{if }SPM\left(i,j\right)\neq 0\\ 0, & else. \end{array}\right. \end{array}$$

Hence, based on (11), we can further obtain a (*D* + *M* + *L*)×(*D* + *M* + *L*) dimensional* Distance Correlation Coefficient Matrix* (*DCCM*) as follows:

(12) $$\begin{array}{c} DCCM\left(i,j\right)=\left\{\begin{array}{cc} \frac{r}{r+1} & \text{if }node\;\;v_{i}=v_{j}\\ P\left(i,j\right), & \text{if }node\;\;v_{j}\in {DCS}_{i}\\ 0, & otherwise \end{array}\right. \end{array}$$

where *i* ∈ [1, *D* + *M* + *L*] and *j* ∈ [1, *D* + *M* + *L*].

**Step 5 (estimation of association degree between a pair of nodes in the disease-miRNA-lncRNA interactive network).**

Based on (12), we can obtain distance correlation coefficient of each nodes pair. For any given nodes pair (*v*_*i*_, *v*_*j*_) in *G* = (*V*, *E*), where *V* = {*d*_1_, *d*_2_,…, *d*_*D*_, *l*_*D*+1_, *l*_*D*+2_,…, *l*_*D*+*L*_} = {*v*_1_, *v*_2_,…, *v*_*D*_, *v*_*D*+1_, *v*_*D*+2_,…, *v*_*D*+*L*_} and {*v*_*i*_, *v*_*j*_}⊆*V*, we can obtain the association degree (AD) between them as follows:

(13) $$\begin{array}{c} AD\left(i,j\right)=\frac{\sum_{kD+M+L}^{k=1}DCCM\left(i,k\right)+\sum_{D+M+L}^{k=1}DCCM\left(k,j\right)}{D+M+L} \end{array}$$

where *i* ∈ [1, *D* + *M* + *L*] and *j* ∈ [1, *D* + *M* + *L*].

**Step 6 (construction of the* Final Prediction Result Matrix*).**

Based on (13), let $AD=\left[\begin{array}{ccc} C_{11} & C_{12} & C_{13}\\ C_{21} & C_{22} & C_{23}\\ C_{31} & C_{32} & C_{33} \end{array}\right]$, where *C*_11_ is a *D* × *D* matrix, *C*_12_ is a *D* × *M* matrix, *C*_13_ is a *D* × *L* matrix, *C*_21_ is a *M* × *D* matrix, C_22_ is a M × M matrix, C_23_ is a M × L matrix, C_31_ is a L × D matrix, C_32_ is a L × M matrix, and C_33_ is a L × L matrix. It can be easily inferred that the matrix C_13_ will be our prediction results, which provided the association probability between each disease and lncRNA. Moreover, we can introduce disease functional similarity and lncRNA functional similarity for C_13_ as follows:

(14) $$\begin{array}{c} FAD=FSD\times C_{13}\times FSL \end{array}$$

where the entity *FAD*(*i*, *j*) in row i column j reflects the probability that the lncRNA *l*(*j*) is related to the disease *d*(*i*).

## 3. Results and Case Studies

To evaluate the prediction performance of DCSLDA, first of all, we implemented LOOCV (leave-one-out cross-validation) to compare DCSLDA with HGLDA [21] based on the lncRNA-disease association dataset downloaded from LncRNADisease database [14]. Next, LOOCV would be implemented to further evaluate the prediction performance of DCSLDA based on the known experimentally verified lncRNA-cancer associations. And then, the effects of the disease functional similarity and the lncRNA functional similarity to the prediction performance of DCSLDA would be analyzed also. Finally, experimental results about the prediction of associations between lncRNAs and three cancers were listed (see Table 1), and the performance comparisons between DCLSDA and HGLDA were implemented according to the rankings of these new disease-related lncRNAs in the case studies of three cancers (see Table 2).

### 3.1. Performance Evaluation of Potential Disease-lncRNA Association Prediction

According to the lncRNA-disease association datasets downloaded from LncRNADisease database, DCSLDA and HGLDA were applied in the framework of LOOCV, respectively. While the LOOCV was implemented for investigated diseases and lncRNAs, each known lncRNA-disease association would be left out in turn as test sample, and then we further evaluated how well this association ranked relatively to the candidate samples. Here, the candidate samples comprised all potential lncRNA-disease pairs without confirmed associations. Therefore, after the implementation of DCSLDA was completed, the rank of each left-out testing sample relative to the candidate samples could be further obtained. And then, the testing samples with a prediction rank higher than the given threshold were considered successfully predicted. Thus, we could further obtain the corresponding true positive rates (TPR, sensitivity) and false positive rates (FPR, 1-specificity) by setting different thresholds. Here, sensitivity refers to the percentage of test samples that were predicted with ranks higher than the given threshold, and the specificity was computed as the percentage of negative samples with ranks lower than the threshold. Therefore, the receiver-operating characteristics (ROC) curves could be drawn by plotting TPR versus FPR at different thresholds. And then, the areas under ROC curve (AUC) would be further calculated to evaluate the prediction performance of DCSLDA. An AUC value of 1 represented a perfect prediction while an AUC value of 0.5 indicated purely random performance.

**Figure 4:**
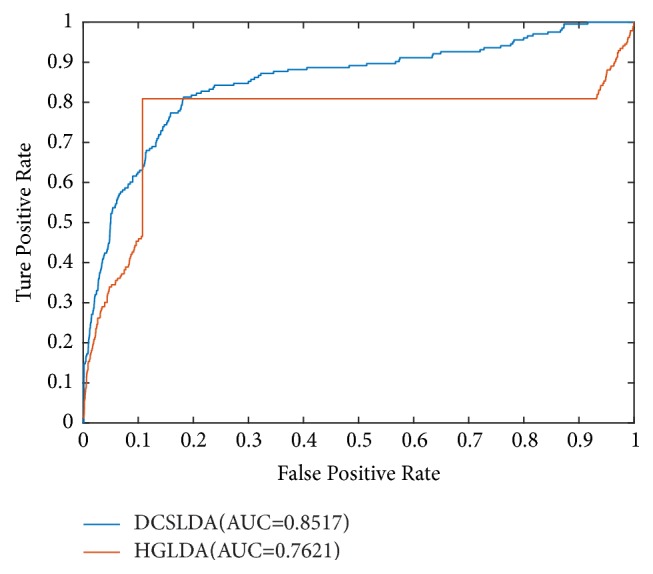
Performance comparisons between DCSLDA and HGDLA in terms of ROC curve and AUC based on LOOCV.

The results of the performance comparison between DCSLDA and HGLDA were shown in Figure 4. Since the HGLDA method predicts lncRNA-disease associations without relying on the information of known disease-lncRNA association, it was selected for performance comparison with our method DCSLDA. As a result, it is clear that our newly proposed method DCSLDA achieved the AUC of 0.8517 in the framework of LOOCV, which is much higher than the AUC of 0.7621 achieved by HGLDA [21]. Simulation results indicate that DCSLDA significantly improved the performance of HGLDA by at least 0.0896 in the term of AUC values and fully demonstrate the performance superiority of HGLDA.

### 3.2. Performance Evaluation of Potential lncRNA-Cancer Association Prediction

**Figure 5:**
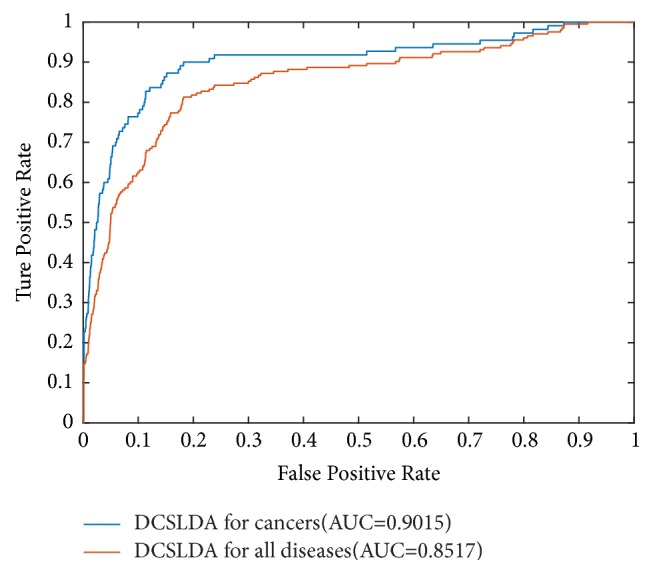
Performance evaluation of potential lncRNA-cancer association prediction in terms of ROC curve and AUC based on LOOCV.

Cancer has become one of the most dangerous killers for human beings [24, 25], and there is a high incidence of cancer in both developed countries and developing countries. Therefore, to further evaluate the prediction performance of DCSLDA, LOOCV was implemented on the basis of 117 lncRNA-cancer associations collected from the LncRNADisease dataset, and the simulation results were illustrated in Figure 5.

From Figure 5, it is easy to find that DCSLDA achieved the AUC of 0.9015 in the frameworks of LOOCV when *r* is set as 6, which indicates that our newly proposed method DCSLDA has a reliable predictive performance of cancers, and therefore it is a precise and high efficient method for the lncRNA-disease association prediction.

### 3.3. Effects of the Disease Functional Similarity and lncRNA Functional Similarity

**Figure 6:**
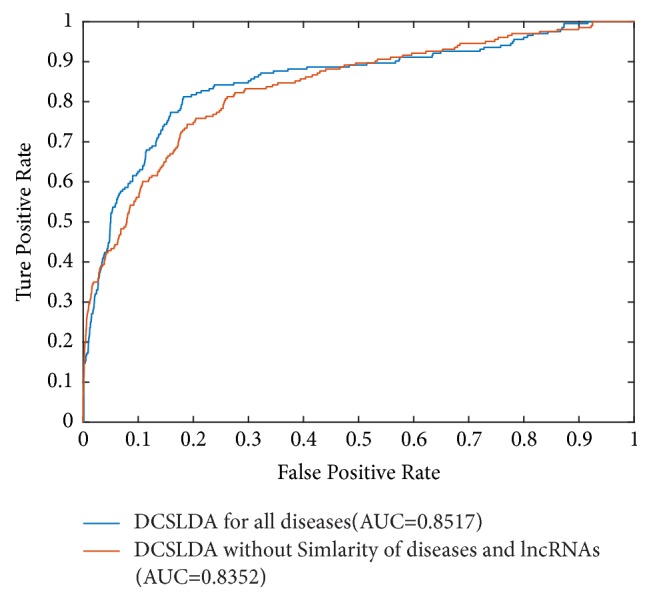
Comparison of effects of the disease functional similarity and lncRNA functional similarity to the prediction performance of PCSLDA in the framework of LOOCV with *r* =6.

In formula (14), we defined  *FAD* = *FSD* × *C*_13_ × *FSL*. Then, in this section, we will analyze the effects of the disease similarity matrix* FSD* and the lncRNA similarity matrix* FSL* through comparing the prediction performances of DCSLDA in the framework of LOOCV while letting *FAD* = *C*_13_ and FAD = FSD × *C*_13_ × *FSL*, respectively. The simulation results are illustrated in Figure 6. It is obvious that DCSLDA achieved the AUCs of 0.8517 while matrixes FSD and FSL were considered, but the AUC achieved by DCSLDA is 0.8352 only when letting FAD = *C*_13_. Simulation results indicated that the prediction performance of DCSLDA will be significantly improved by introducing the similarity matrixes FSD and FSC. Moreover, in Table 1, DCSLDA was applied to three important kinds of cancer (breast cancer, colorectal cancer, and lung cancer). As a result, 17 predicted lncRNA-disease pairs with high predicted value were publicly released to benefit the biological experimental validation.

### 3.4. Case Studies

Obviously, DCSLDA can predict all potential relationships between diseases and lncRNAs in* dataset1* and* dataset2* simultaneously. And of course, potential associations with high predicted value can be publicly released to benefit the biological experimental validation. It is anticipated that these potential disease-lncRNA associations that significantly share common miRNAs could be validated by biological experiments and provide important complement for experimental studies. Moreover, plentiful evidence has indicated that lncRNAs played important roles in various kinds of human cancers. The predicted results were sorted from best to worse, among which the first 0.5% results are selected to be analyzed (see Supplementary file 4). Case studies about three important kinds of cancers based on top 0.5% of predicted results were implemented to show the predictive performance of DCSLDA. Prediction results were verified based on the recent updates in the LncRNADisease dataset and recently published experimental literature (ranking results have been listed in Table 1).

In the world, breast cancer is the most prevalent cancer in women and a major public health problem. Several studies have focused on studying this disease, but more are needed, especially at the genetic and molecular levels [26, 27]. Therefore, it is necessary to predict breast cancer-related lncRNAs and identify lncRNA biomarkers. DCSLDA was implemented to prioritize candidate lncRNAs for breast cancer. Among the first 5% of predictive results, nine breast cancer-related lncRNAs have been confirmed based on recent experimental literature (see Table 1). For example, KCNQ1OT1, MALAT1, XIST, and NEAT1 are experimentally confirmed breast cancer-related lncRNAs, which have been ranked 2nd, 11th, 12th, and 19th in the predicted list based on the model of DCSLDA, respectively. KCNQ1OT1 had significantly higher expression levels in invasive breast carcinoma and was induced by estrogen in estrogen receptor-alpha expressing breast cancer cells [28]. 17*β*-Estradiol treatment affects breast tumor or nontumor cells proliferation, migration, and invasion in an ER*α*-independent, but a dose-dependent, way by decreasing the MALAT1 RNA level [29]. XIST expression is significantly reduced in breast cancer cell lines and breast cancer samples [30]. Breast cancer patients with high level of NEAT1 expression show low survival rate [31].

Colorectal cancer (CRC) is a leading cause of cancer deaths worldwide, one of the fundamental processes driving the initiation and progression of CRC is the accumulation of a variety of genetic and epigenetic changes in colon epithelial cells. Colorectal cancer is usually caused by the combination of various factors, such as genetic and epigenetic changes [32, 33]. Specially, lncRNAs have been demonstrated to play a critical role in the development and progression of colon cancer [34]. As a result, six colorectal cancer-related lncRNAs were listed in Table 1. For example, Tanaka K et al. proved that Loss of imprinting of KCNQ1OT1 is considered as a useful marker for diagnosis of colorectal cancer because of its frequent occurrences in colorectal cancer samples [35]. Ji Q et al. findings implied that MALAT1 might be a potential predictor for tumor metastasis and prognosis [36]. Furthermore, the interaction between MALAT1 and SFPQ could be a novel therapeutic target for CRC. Lassmann S et al. proved that expression level change of or DNA amplification of XIST is associated with colorectal cancer [37].

Over the past 30 years, the morbidity and mortality of lung cancer have been increasing and the cancer has the highest incidence and mortality across the world [38]. Due to the early diagnosis of lung cancer and the lack of effective treatment, its survival rate is around 10% within five years, which seriously endangers human health. More and more evidence has shown that lncRNAs play a critical role in treatment of lung cancers. Among the first 5% of predictive results, three predicted lncRNAs have been confirmed by published experimental literature [39]. According to this literature, MALAT1 has been shown to be highly associated with metastasis of lung cancer and promote lung cancer cell motility by regulating motility related gene expression [40, 41]. Long noncoding RNA XIST acts as an oncogene in non-small cell lung cancer by epigenetically repressing KLF2 expression [42].

In addition, performance comparisons between DCSLDA and HGLDA were implemented according to the rankings of these disease-related lncRNAs in the case studies of breast cancer, colorectal cancer, and lung cancer (see Table 2). By ranging the predicated results by HGLDA and our methods from good to bad, we selected the intersection of the underlying disease-lncRNA relationship predicated by HGLDA and the first 0.5 percent of the predicted results by our methods and listed the lncRNA items related to breast cancer, colorectal cancer, and lung cancer in this intersection in Table 2. As a result, DCSLDA significantly improved the prediction ability of HGLDA with higher ranks for these new disease-related lncRNAs.

## 4. Discussion and Conclusions

In recent years, plenty of studies have generated an enormous amount of biological data related to lncRNAs. Accumulating evidence shows that lncRNAs have played a very important role in the biological functions, and the study of lncRNA-disease association prediction is of great significance to human beings. However, there is a few computational models for predicting potential disease-lncRNA associations based on the information of miRNA. To utilize the wealth of disease-miRNA, miRNA-lncRNA, and disease-lncRNA associations data collected from three datasets and recently published in experimental literature, in this article, the novel model of DCSLDA was developed to predict potential disease-lncRNA associations. We calculated distance correlation set of each node based on disease-miRNA-lncRNA interactive network first and then further integrated disease functional similarity and lncRNA functional similarity for DCSLDA. The important difference from previous computational model is that DCSLDA does not rely on any known disease-lncRNA associations and it predicts disease-lncRNA associations only based on disease-miRNA-lncRNA interactive network. In order to evaluate the prediction performance of DCSLDA, the validation frameworks of LOOCV were implemented based on known disease-lncRNA and cancer-related-lncRNA associations downloaded from LncRNADisease database. And case studies were further implemented to three important cancers (breast cancer, colorectal cancer, and lung cancer) based on recently published experimental literature. The simulation results show that DCSLDA can achieve reliable and excellent prediction performance and is superior to the state-of-the-art methods. Hence, it is anticipated that DCSLDA could play an important role in the prospective biomedical researches.

Disease functional similarity plays an important role in disease-related molecular function research. Functional associations between disease-related genes are often used to identify pairs of similar diseases from different perspectives. Calculating lncRNA functional similarity could benefit lncRNA function inference and disease-related lncRNA prioritization. Therefore, based on the two assumptions that (1) similar diseases tend to show a similar interaction and noninteraction pattern with the miRNAs and (2) similar lncRNAs tend to show a similar interaction and noninteraction pattern with the miRNAs, DCSLDA was developed to predict potential disease-related lncRNA by integrating lncRNA functional similarity and disease functional similarity. Simulation results indicated that the prediction performance of DCSLDA will be significantly improved by disease similarity and lncRNA similarity.

However, there are also some limitations in our method. Firstly, DCSLDA measures the correlations between lncRNAs and investigated diseases by integrating walks with different lengths in a lncRNA-miRNA-disease network, which is constructed by combining the known disease-miRNA network, miRNA-lncRNA network, and disease similarity network. The value of distance threshold parameters* r *is an important factor in DCSLDA, and how to select this parameter is not yet solved well. Secondly, although DCSLDA does not rely on any known experimentally verified lncRNA-disease relationships, the performance of DCSLDA was not very satisfactory compared with that of several existing methods. In the future, we will further integrate data of diseases and lncRNAs that do not rely on the lncRNA-disease interactive network, disease-miRNA interactive network, or miRNA-lncRNA interactive network; then these above problems may be well solved. Finally, introducing more reliable measure of disease similarity and lncRNA similarity and developing more reliable similarity integration method would improve the performance of DCSLDA. In particular, disease similarity and lncRNA similarity in this model totally rely on known disease-miRNA and miRNA-lncRNA associations. The performance of DCSLDA would be further improved when sequence similarity of lncRNA and semantic similarity of disease are introduced.

## Figures and Tables

**Table 1:** 17 predicted lncRNA-disease pairs with high predicted value while DCSLDA was applied to three important kinds of cancer (breast cancer, colorectal cancer, and lung cancer).

Cancer	LncRNA	PMID
Breast cancer	KCNQ1OT1	21304052; 26323944

Breast cancer	MALAT1	24525122; 19379481

Breast cancer	XIST	27248326

Breast cancer	NEAT1	25417700; 28034643

Breast cancer	LINC00657	26942882

Breast cancer	SNHG16	28232182

Breast cancer	CASP8AP2	28388918

Breast cancer	PPP1R9B	26387546

Breast cancer	TUG1	27791993

Colorectal cancer	KCNQ1OT1	16965397; 11340379

Colorectal cancer	MALAT1	25025966

Colorectal cancer	XIST	17143621

Colorectal cancer	NEAT1	26552600

Colorectal cancer	SNHG16	26823726

Colorectal cancer	CASP8AP2	22216762

Lung cancer	MALAT1	20937273; 24757675; 24667321

Lung cancer	XIST	27501756

**Table 2:** Performance comparisons between DCSLDA and HGLDA based on the rankings of ten lncRNA-disease associations related to three important kinds of cancer (breast cancer, colorectal cancer, and lung cancer).

Cancer	LncRNA	DCSLDA	HGLDA
Breast cancer	KCNQ1OT1	1	8

Breast cancer	MALAT1	4	30

Breast cancer	XIST	5	1

Breast cancer	NEAT1	8	12

Breast cancer	SNHG16	12	3

Colorectal cancer	KCNQ1OT1	1	5

Colorectal cancer	MALAT1	4	3

Colorectal cancer	XIST	5	1

Lung cancer	MALAT1	4	9

Lung cancer	XIST	5	1

Average ranks		4.9	7.3

## Supplementary Materials

Supplementary Materials Supplementary file 1: the known miRNA-disease associations for constructing the ASC1. We list 5430 known miRNA-disease associations which were collected from HMDD dataset to construct the ASC1. Supplementary file 2: the known lncRNA-miRNA associations for constructing the ASC2. We list 10195 known lncRNA-miRNA associations which were collected from starBase v2.0 database to construct the ASC2. Supplementary file 3: the known lncRNA-disease associations. We list 203 high-quality lncRNA-disease associations which were collected from LncRNADisease database to validate the performance of our method. Supplementary file 4: the top 0.5% results were listed to validate the performance of our method.
